# Microplastics as a Modifier of Polycyclic Aromatic Hydrocarbon (PAH) Toxicity: A Review on Context-Dependent Effects Across Organisms

**DOI:** 10.3390/biology15060455

**Published:** 2026-03-11

**Authors:** Cris Gel Loui A. Arcadio, Jay Rumen U. Maglupay, Andros M. Po, Jhosin Jaik B. Pardillo, Hernando P. Bacosa

**Affiliations:** 1 Academic Unit, Mindanao State University-Main Campus Bataraza Extension (MSU-MCBE), Inogbong, Bataraza 5306, Palawan, Philippines; crisgelloui.arcadio@g.msuiit.edu.ph; 2 College of Education, Mindanao State University-Main Campus, Marawi City 9700, Lanao del Sur, Philippines; 3 Department of Environmental Science, School of Interdisciplinary Studies, Mindanao State University-Iligan Institute of Technology, A. Bonifacio Avenue, Tibanga, Iligan City 9200, Philippines; jayrumen.maglupay@g.msuiit.edu.ph (J.R.U.M.); andros.po@g.msuiit.edu.ph (A.M.P.); jhosinjaik.pardillo@g.msuiit.edu.ph (J.J.B.P.); 4 Environmental Pollution, Innovation and Circularity Laboratory, Mindanao State University-Iligan Institute of Technology, A. Bonifacio Avenue, Tibanga, Iligan City 9200, Philippines

**Keywords:** microplastics, polycyclic aromatic hydrocarbons, co-exposure, oxidative stress, conserved stress pathways, context-dependent toxicity

## Abstract

Microplastics and polycyclic aromatic hydrocarbons frequently co-occur in aquatic and terrestrial ecosystems, where their combined biological effects remain incompletely understood. Although both stressors exhibit well-documented individual toxicities, co-exposure studies report highly variable outcomes, ranging from enhanced or reduced toxicity to neutral responses. This review synthesizes findings from 45 peer-reviewed studies examining single and combined microplastic–PAH exposures across aquatic vertebrates, invertebrates, plants, microorganisms, and cell-based systems. Rather than introducing novel toxic mechanisms, microplastics primarily modulate the probability, magnitude, and timing of conserved biological response pathways. Across taxa, oxidative stress, metabolic disruption, immune modulation, developmental impairment, and behavioral alterations emerge as recurrent endpoints, with responses strongly shaped by context. Particle size, polymer type, exposure concentration and duration, and organismal traits consistently determine whether microplastics enhance PAH bioavailability, reduce effective exposure through sorption, or result in mixed or negligible effects. Overall, the evidence indicates that microplastics function as dynamic modifiers of chemical stress rather than universal toxicity amplifiers. These findings underscore the limitations of single-contaminant risk frameworks and highlight the need for biology-centered, mixture-based approaches that account for exposure pathways, life-history traits, and conserved stress-response systems in ecological risk assessment.

## Simple Summary

Microplastics and toxic chemicals called polycyclic aromatic hydrocarbons are now found together in water, soil, and living organisms. Scientists are concerned because plastics can carry these chemicals and may change how harmful they are to plants and animals. However, past studies have reported different results, making it unclear whether plastics always make chemical pollution more dangerous. This review aimed to understand why these differences occur by examining how living organisms respond when exposed to plastics and these chemicals at the same time. By comparing studies across fish, plants, and microorganisms, we found that plastics do not create new types of harm but instead change how often and how strongly normal stress responses in living organisms are triggered. Sometimes plastics increase harm by helping chemicals enter the body, while in other cases they reduce harm by trapping chemicals and limiting exposure. The outcome depends on the size and type of plastic, how exposure occurs, and the traits of the organism. These findings help explain conflicting results in earlier studies and support more realistic approaches to studying pollution. This knowledge is valuable to society because it improves how environmental risks are assessed and supports better decisions to protect ecosystems and human health.

## 1. Introduction

Plastic materials have become integral to modern society, with global production increasing steadily over recent decades. Because of their persistence and fragmentation, plastics accumulate in the environment as microplastics (MPs; <5 mm), which are now widely distributed across marine [1,2], freshwater [3,4,5,6], and terrestrial ecosystems [7] and organisms inhabiting them [2,4,8]. These particles have been found in a variety of habitats, from urban rivers to isolated locations such as arctic areas, deep-sea sediments, and high-altitude lakes, demonstrating their global ecological significance [9,10]. At the same time, polycyclic aromatic hydrocarbons (PAHs) are still among the most common organic pollutants worldwide. PAHs, which come from both petrogenic and pyrogenic sources, persist in environmental matrices and are well known for inducing oxidative stress, disrupting endocrine and metabolic systems, impairing development, and causing genotoxic and carcinogenic consequences in a variety of animals [11,12].

MPs and PAHs are typically found together in environments with significant biological activity, such as sediments, soils, and productive aquatic systems [13,14]. Although much of the early literature has focused on aquatic environments, microplastic–PAH interactions are also increasingly recognized in terrestrial systems, including soils and plant-based exposure pathways, where particle properties, soil matrices, and root–environment interactions can substantially influence contaminant bioavailability and biological responses. MPs have hydrophobic surfaces and large specific surface areas, which allow them to sorb PAHs and other organic pollutants, affecting their environmental partitioning and exposure paths [15]. Early research saw microplastics primarily as passive carriers that improve pollutant transport and bioavailability, raising fears that polymers generally increase chemical toxicity [16,17]. However, rising experimental evidence challenges this notion. In contrast, co-exposure studies show very diverse biological outcomes, including synergistic toxicity, antagonistic or reduced effects, and responses that are indistinguishable from single-contaminant exposure [18,19].

From a biological standpoint, this variety indicates that microplastic–PAH interactions do not bring fundamentally new forms of toxicity. Rather, they alter how organisms respond to chemical stress by adjusting internal dose, exposure timing, and route of uptake. PAHs generally cause toxicity across taxa via conserved biological pathways, such as the production of reactive oxygen species, activation of detoxification systems, disruption of mitochondrial function, interference with developmental signaling, and modulation of immunological responses [20]. MPs indirectly affect these pathways by influencing the frequency and amount of PAHs that reach sensitive tissues and cells [21]. Whether this results in enhanced, diminished, or neutral biological impacts is determined by a mix of particle properties, ambient factors, and organismal attributes.

Previous reviews have made significant contributions to understanding the interactions between plastics and organic contaminants, notably in terms of sorption mechanisms, polymer chemistry, and environmental destiny [22]. While these studies give important context, many are primarily concerned with physicochemical interactions and environmental movement, with little attention on how cumulative exposures are integrated at the cellular, physiological, and organismal levels. Furthermore, studies of single-contaminant and co-exposure effects across equivalent biological endpoints are lacking, making it difficult to generalize how MPs affect biological responses to PAHs.

This review aims to summarize current knowledge on the ecotoxicological consequences of MPs, PAHs, and their combined exposure in aquatic and terrestrial systems. Although MPs interact with a broad spectrum of organic contaminants, this review focuses on PAHs (PAHs) because of their global environmental prevalence, persistence, and well-established toxicological profiles. PAHs co-occur extensively with MPs in sediments, soils, and aquatic systems and exhibit strong sorption affinity for common polymers, making them a robust model group for investigating microplastic-mediated exposure modification. Moreover, PAH toxicity is mediated through conserved biological pathways across taxa, enabling cross-system comparison of biological responses under co-exposure scenarios. The extensive use of PAHs in experimental microplastic co-exposure studies further allows systematic synthesis of context-dependent outcomes across organisms, exposure routes, and particle properties. By combining findings from studies on vertebrates, invertebrates, plants, microbes, and cell-based models, this review finds shared biological response patterns and critical elements that influence context-dependent outcomes. It specifically investigates how particle size, polymer type, exposure conditions, and organismal features interact to influence the activation, intensity, and timing of conserved stress response pathways. This review aims to clarify inconsistencies in the literature by framing microplastic–PAH interactions as modifiers of biological stress integration rather than uniform toxicity drivers, and to contribute to more biologically relevant approaches in ecotoxicological research and environmental risk assessment.

## 2. Methodology

### Recovery of Suitable Articles

A literature-based review was conducted to synthesize peer-reviewed studies examining interactions between plastics (including micro- and nanoplastics) and PAHs in environmental and biological systems. Google Scholar was used as the primary search engine to identify relevant studies. To ensure broader coverage and reduce the risk of missing key publications, additional academic databases were also consulted, including Scopus (Elsevier) and PubMed (U.S. National Library of Medicine). The Web of Science (WoS) database was not included in the search strategy due to institutional access limitations. The use of multiple databases in the present review is consistent with Vo & Pham’s (2021) [23] review on microplastics and associated contaminants. In addition, bibliometric evidence indicates that Google Scholar captures nearly all citations indexed in WoS and Scopus while providing broader coverage of interdisciplinary and emerging research fields [24].

Searches were performed using direct keywords and phrases related to plastics, contaminants, and ecological effects, including plastic, microplastic, nanoplastic, polycyclic aromatic hydrocarbons, PAHs, toxicity, co-exposure, bioaccumulation, and ecological risk. Titles and abstracts of retrieved records were initially screened to assess their relevance, followed by full-text evaluation of potentially eligible studies. The following criteria were applied to ensure that selected studies were relevant: 1. Published as peer-reviewed original research articles. 2. Specifically investigated micro- or nano-plastics and PAHs under co-exposure conditions. 3. Involved environmental matrices or biological systems (organisms or cell lines). 4. Reported sufficient experimental or field data, including plastic size, polymer type, PAH identity, or exposure concentration. Studies that did not meet these criteria were excluded. Also, the gray literature, such as government reports, technical documents, policy papers, and academic theses, and other unpublished materials were not included in the screening process.

**Figure 1:**
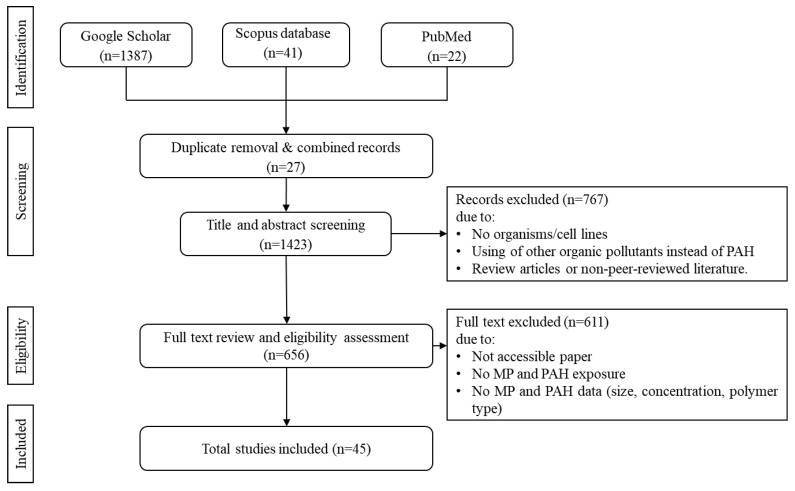
PRISMA Flow diagram of the selection of published studies, including the process of literature identification and eligibility criteria.

A total of 45 peer-reviewed articles were included in this review (Figure 1). Key information was systematically extracted using Microsoft Excel 2024, including author names, year of publication, environmental system or organism examined, plastic type and size, PAH type, exposure conditions, main findings, and conclusions. Studies were then grouped into thematic categories based on polymer type, PAH compound, environmental context, biological system, and the nature of plastic–PAH interactions to support cross-study comparison and synthesis. The additional references were included in the present review to provide background information and context to support the discussions and to strengthen the narrative and interpretation of the results.

## 3. Research Trends in Microplastic–PAH Co-Exposure Studies

**Figure 2:**
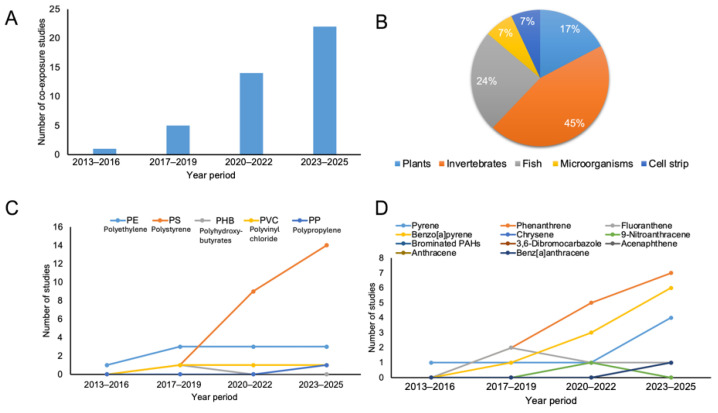
Overview of research trends in microplastic–polycyclic aromatic hydrocarbon co-exposure studies, showing (**A**) the increase in published studies over time, (**B**) the distribution of biological test systems, (**C**) commonly used plastic polymer types in studies, and (**D**) frequently studied chemical compounds.

Over the last decade, there has been a significant increase in research on the combined biological impacts of MPs and PAHs, reflecting an increasing recognition that organisms in natural habitats are exposed to multiple stressors simultaneously rather than separately. As illustrated in Figure 2A, early research before to 2016 was restricted, with only a few co-exposure studies available. Since 2017, there has been a steady increase in research output, followed by a significant acceleration beyond 2020. This expansion corresponds to broader trends in biological and environmental sciences toward mixture toxicity, mechanistic integration, and ecologically relevant exposure scenarios. The recent rise in publications reflects a growing recognition that single-contaminant studies are insufficient to understand how living systems respond to complex chemical and particle stresses.

The biological models used in co-exposure studies are not evenly distributed among taxa (Figure 2B). Invertebrates make up most test organisms, accounting for over half of all research, followed by fish. These models are preferred because they are ecologically realistic, experimentally tractable, and respond to changes in oxidant balance, immunological function, and development [25]. Plants, microbes, and cell-based systems are underrepresented, while playing critical roles in ecosystem processes and pollutant transformation [26]. This taxonomic bias limits our understanding of how microplastic–PAH interactions affect primary producers, microbial ecosystems, and fundamental biological processes, revealing a significant gap in future biological study.

The use of plastic materials in co-exposure studies also reflects methodological convenience rather than environmental representativeness (Figure 2C). Polystyrene is by far the most widely used polymer, especially in recent years, owing to its commercial availability in uniform particle sizes and great affinity for hydrophobic compounds [27,28]. In contrast, polyethylene and polypropylene, which dominate plastic production and environmental abundance, receive far less attention likely because these polymers are less available in standardized, uniform particle sizes and shapes, making them more challenging to use in controlled experimental studies compared with commercially available polystyrene spheres. This imbalance may influence reported toxicity patterns because polymer type influences particle absorption, tissue contact, and chemical release. Extending investigations to include environmentally dominating polymers and weathered plastics will be critical for increasing the biological relevance of experimental results.

A similar pattern emerges in the selection of chemical substances (Figure 2D). A small number of PAHs, including phenanthrene, pyrene, and benzo[a]pyrene, dominate co-exposure studies. These substances are frequently employed as model chemicals because their biological effects, such as oxidative stress induction and metabolic disturbance, have been widely studied [29,30]. While this strategy improves study comparability, it hinders understanding of how organisms respond to more complex and realistic chemical mixes. As a result, most studies concentrate on short-term physiological and cellular endpoints, with fewer investigations relating co-exposure impacts to long-term biological outcomes like growth, reproduction, and population-level fitness.

Taken together, the trends summarized in Figure 2 reveal that current knowledge on microplastic–PAH co-exposure effects is shaped by strong biases in study design, organism selection, and material choice. While these studies have substantially advanced understanding of conserved biological stress responses, they also underscore the need for broader taxonomic coverage, greater material diversity, and biologically integrated endpoints. Addressing these gaps will be critical for developing a more comprehensive understanding of how living organisms integrate combined particulate and chemical stress under environmentally realistic conditions.

## 4. Environmental Processes Shaping Biologically Relevant Exposure

### 4.1. MPs as Biologically Active Exposure Modifier

MPs affect biological exposure not just by transferring adsorbed chemicals, but also by altering how organisms interact with those chemicals. Particle size and polymer type influence ingestion, particle retention, and tissue contact, all of which influence biological uptake and response.

#### 4.1.1. Particle Size-Dependent Interactions

Smaller particles are more likely to interact directly with the epithelium surface and be consumed by a range of organisms [31]. In certain systems, the capacity of small particles to cross biological barriers or be absorbed by cells through endocytic pathways increases the likelihood of the associated compounds reaching intracellular targets [32]. Conversely, larger particles can be rapidly consumed and usually remain within the intestinal lumen, thereby reducing internal exposure. These size-dependent interactions help explain why different biological outcomes are often observed in studies using similar chemical doses but different particle sizes [33].

#### 4.1.2. Surface Chemistry and Particle Retention

Surface chemistry has a significant role in determining how MPs interact with biological tissues. Aging and weathering can increase surface roughness and provide functional groups that improve biological adhesion and chemical binding [34]. These modified surfaces may increase the retention time or promote interaction with mucus layers, epithelial cells, and immune-responsive tissues in organisms. The dynamics of intake and retention significantly influence the biological effects of microplastic exposure. Ingested particles can act as localized sources of chemical exposure in the digestive tract when conditions there promote desorption. Extended retention increases the risk of cumulative stress, which can influence the duration of exposure [35]. Crucially, gut-associated exposure alone can cause systemic physiological reactions through stress signaling, immunological activation, and metabolic disturbance; these mechanisms do not require MPs to penetrate interior tissues [36].

#### 4.1.3. Biological Feedback and Exposure Modulation

Chemical exposure may be influenced by interactions between MPs and biological defense mechanisms. Contact with epithelial surfaces can compromise barrier integrity or trigger immune-like reactions, potentially increasing chemical permeability. Simultaneously, organisms may respond to particle exposure by upregulating antioxidant and detoxification pathways, altering how subsequent chemical stress is handled. These interactions reinforce the function of MPs as active exposure modifiers by highlighting the dynamic feedback between physical particles and biological control.

Altogether, these pathways show how MPs alter exposure by altering the temporal and spatial distribution of chemical stress in organisms. By changing the likelihood, severity, and duration of physiologically relevant exposure, MPs introduce unpredictability rather than serving as consistent amplifiers or mitigators of toxicity. This viewpoint highlights the significance of assessing microplastic–chemical interactions through a biological lens focused on organismal integration rather than ambient concentration alone and explains why co-exposure effects are very context dependent.

### 4.2. Sorption–Desorption Dynamics Within Biological Systems

Understanding context-dependent toxicity requires an understanding of sorption–desorption mechanisms, which control how MPs affect the biologically effective dose of related compounds. The biological importance of sorption lies in its role in modifying internal exposure when particles interact with organisms, even though it is often described in terms of environmental partitioning. From a biological standpoint, sorption–desorption dynamics dictate when, where, and how chemicals are delivered to delicate tissues, rather than whether they are present.

Because of their surface characteristics, MPs readily bind hydrophobic compounds, thereby reducing the fraction of freely dissolved molecules in nearby water or soil. In certain situations, this sequestration may lessen short-term biological impacts and acute external exposure [22]. However, this seeming alleviation is conditional and frequently fleeting from a biological perspective. Instead of being eliminated from the system, sorbed chemicals are dispersed and retained on particles that may eventually enter biological compartments [37]. Therefore, rather than lasting detoxification, sorption should be understood as a delay or rerouting of exposure. Once MPs are ingested or come into close contact with biological tissues, chemical release is influenced by internal conditions that differ markedly from those of the external environment [35]. Digestive fluids, lipid-rich tissues, enzymatic activity, and variable pH can all promote desorption of bound chemicals [38]. This internal release increases chemical concentrations at biological interfaces, effectively shifting exposure from the environment to within the organism. Significantly, this process alters exposure timing and internal dose without changing the chemical’s intrinsic toxic properties, explaining why co-exposure effects often differ from predictions based solely on environmental concentrations. Furthermore, desorption may directly affect immune cells, epithelial tissues, and associated bacteria, especially environmental MPs, which can induce a strong inflammatory response in various human cells and tissues [39]. Therefore, spatially concentrated exposure rather than uniform distribution may result in biological effects, underscoring the significance of taking tissue-specific exposure dynamics into account when evaluating co-exposure research.

Sorption–desorption is a dynamic equilibrium that is impacted by particle properties, chemical parameters, and biological environment rather than being a one-way process. Chemical hydrophobicity, surface aging, and particle size all affect how easily chemicals attach to and are released from MPs. This balance can change quickly in biological systems when particles travel between organisms and environmental compartments. Therefore, depending on when and where desorption takes place in relation to delicate physiological processes, the same exposure conditions can result in various biological effects.

Understanding sorption–desorption as a process that modifies internal exposure helps to explain why co-exposure results are so inconsistent and emphasizes the importance of interpreting microplastic–chemical interactions using internal dose and exposure time rather than just environmental concentration.

### 4.3. Exposure Pathways Shaped by Organismal Ecology and Life History

Organismal features, including feeding behavior, habitat use, and developmental organismal ecology and life-history characteristics, play a vital role in determining biologically relevant exposure to MPs and related pollutants. Microplastic-mediated exposure is dependent on how organisms interact with particles in their surroundings, in contrast to simply chemical exposures [40], which may be approximated by external concentrations. Whether exposure happens mainly by ingestion, surface contact, or indirect trophic transfer depends on feeding strategy, habitat utilization, and developmental stage. These pathways have a significant impact on internal dose and biological response.

#### 4.3.1. Feeding Strategy and Habitat Use

One of the main factors influencing the ingestion of MPs and subsequent chemical exposure is the feeding mode [41]. Large amounts of water are continuously processed by filter feeders and suspension feeders, which raises the possibility of particle absorption and extended gut exposure to sorbed chemicals [42]. Benthic invertebrates and deposit feeders come into contact with MPs linked to sediments [2], where particles may have increased chemical loads because of extended environmental exposure. Instead of coming into direct contact with the environment, predatory species may be indirectly exposed through trophic transfer, meeting MPs and related substances through their prey [43]. The large variations in ingestion rates and biological consequences among taxa under comparable environmental conditions can be explained by these variations in feeding strategies.

#### 4.3.2. Life History Stage and Physiological Sensitivity

Another factor influencing biological vulnerability to co-exposure is life history stage. Due to rapid cell division, inadequate barrier formation, and limited detoxification capacity, early developmental phases often exhibit increased sensitivity. Even slight increases in internal chemical exposure throughout these phases can cause long-lasting physiological alterations or interfere with developmental processes [44]. Adult organisms, on the other hand, might have stronger detoxification systems, although long-term exposure could have cumulative consequences [45]. The significance of exposure timing in determining biological outcomes is highlighted by this life-stage dependence.

#### 4.3.3. Internal Processing and Species-Specific Responses

Physiological traits such as gut morphology, digestive efficiency, and metabolic rate influence how ingested MPs and associated chemicals are processed within organisms [46]. Variations in metabolic capability govern how well chemicals are converted or removed, whereas differences in intestinal residence length impact the chance for chemical desorption [35]. Even among creatures that occupy similar ecological niches, these internal processing characteristics contribute to species-specific exposure profiles.

The biological framework of exposure is determined by the combination of feeding behavior, habitat utilization, life-history stage, and physiological characteristics. Instead of functioning as consistent stressors, MPs interact with this framework by changing the path, intensity, and duration of chemical exposure. As a result, how organisms incorporate particle-mediated exposure into their preexisting physiological frameworks is reflected in their biological reactions to co-exposure. This viewpoint highlights the necessity of organism-centered approaches in co-exposure research and explains why identical co-exposure settings might result in different outcomes across species and life stages.

### 4.4. Temporal Dynamics and Chronic Low-Level Stress

Time is an essential yet often overlooked factor in microplastic–PAH exposure. Stress does not cause biological systems to react instantly or consistently; instead, responses develop gradually as organisms integrate exposure, trigger defense mechanisms, and try to recover physiologically. By changing the timing, length, and frequency of chemical exposure, MPs affect these temporal dynamics and the way stress-response pathways are activated. Short-term exposures frequently trigger stress-response mechanisms, including detoxification and antioxidant defenses, to activate rapidly. If particles are ingested soon or if there is negligible desorption of related compounds, MPs may have little effect on total toxicity under these severe circumstances [47]. On the other hand, prolonged exposure raises the possibility that MPs may be regularly consumed or stored, which permits the long-term release of related compounds. Even at comparatively low external quantities, this prolonged exposure can change biological reactions from short-term stress to long-term physiological adjustment or malfunction [48].

### 4.5. Environmental Aging, Biofilms and Biological Availability

Once released into the environment, MPs rarely retain their pristine physicochemical properties. Instead, they undergo continuous environmental aging driven by ultraviolet radiation, mechanical abrasion, temperature fluctuations, and oxidative and microbial processes. These transformations alter surface roughness, functional group composition, hydrophobicity, and charge, thereby reshaping interactions between MPs, PAHs (PAHs), and biological systems.

Environmental aging generally increases surface oxidation and porosity, which can enhance the sorption capacity of MPs for hydrophobic organic contaminants such as PAHs [49]. However, increased sorption does not necessarily translate into increased biological toxicity. Aging can simultaneously reduce desorption rates, limiting PAH bioavailability and attenuating biological uptake under certain exposure scenarios [50]. As a result, aged MPs may function either as sinks that reduce effective chemical exposure or as delayed-release reservoirs, depending on exposure duration, gut residence time, and surrounding chemical gradients.

In natural environments, aged MPs are rapidly colonized by microorganisms, forming complex biofilms that further modify contaminant dynamics [51]. Conversely, biofilm formation may enhance biological availability by increasing particle accessibility, facilitating ingestion by invertebrates and vertebrates, and prolonging particle retention within digestive systems. In such cases, MPs may act as vectors that locally concentrate PAHs and deliver them to internal tissues, particularly in biofilms, which can sequester PAHs through microbial uptake, extracellular polymeric substances, and metabolic transformation, thereby reducing freely available PAHs at the microplastic surface in organisms with limited detoxification capacity.

Importantly, biofilms are biologically active interfaces rather than passive coatings. Microbial communities associated with MPs can alter PAH composition through partial biodegradation, producing metabolites with distinct toxicological profiles. These transformations may shift biological responses from acute toxicity toward sublethal effects such as oxidative stress, immune modulation, and metabolic disturbance. Consequently, observed biological outcomes often reflect integrated responses to particles, parent PAHs, and biofilm-mediated transformation products rather than exposure to a single stressor [52].

Collectively, environmental aging and biofilm formation introduce substantial variability into microplastic–PAH interactions by modifying both chemical availability and biological exposure pathways. These processes help explain why laboratory studies using pristine particles frequently overestimate or mischaracterize ecological risks. Incorporating environmentally aged and biofilm-coated MPs into experimental designs is therefore essential for accurately resolving the context-dependent nature of microplastic–PAH co-exposure and its biological consequences.

## 5. Ecotoxicological Effects of MPs, PAHs and Their Co-Exposure

### 5.1. Aquatic Vertebrates

Table 1 summarizes various scientific studies investigating the toxicological impacts of MPs (MPs) and PAHs (PAHs). In aquatic vertebrates, particularly fish, MPs and PAHs have been extensively investigated, with zebrafish (*Danio rerio*), barramundi (*Lates calcarifer*), marine medaka (*Oryzias melastigma*), and goldfish (*Carassius auratus*) emerging as model organisms.

#### 5.1.1. Developmental and Physiological Toxicity in Fish

Multiple studies expose zebrafish embryos or larvae to polystyrene (PS) nanoparticles (55–100 nm) co-occurring with PAHs such as chrysene, fluoranthene, phenanthrene, benz[a]anthracene, and brominated PAHs [57,59,67,69,70]. Early developmental stages are particularly vulnerable to PAHs due to their reliance on rapid cell division and tightly regulated gene expression, making zebrafish embryos highly sensitive indicators of subtle toxic interactions [71]. Zebrafish studies consistently report oxidative stress, developmental abnormalities, cardiotoxicity, neurotoxicity, and cellular damage when exposed to polystyrene (PS) MPs or nanoparticles in combination with PAHs such as phenanthrene, chrysene, fluoranthene, benz[a]anthracene, and nitrated or brominated PAHs [57,59,67,70,72]. Meanwhile, smaller MPs and nanoparticles (≤1 µm) significantly intensified toxicity, enhancing PAH uptake and physiological disruption. However, in some cases, adsorption of PAHs onto MPs reduced freely dissolved PAH concentrations, thereby mitigating DNA damage and genotoxicity [59,73].

#### 5.1.2. Behavioral and Gut-Associated Effects

Juvenile *Lates calcarifer* exposed to polyethylene (PE) or PS MPs with pyrene or benzo[a]pyrene showed reduced feeding efficiency and impaired swimming behavior, indicating that behavioral toxicity is a sensitive endpoint [65,66]. Marine medaka (*Oryzias melastigma*) studies further demonstrated gut-level toxicity, microbiome dysbiosis, and metabolic disruption, suggesting that MP–PAH interactions may have long-term and potentially transgenerational consequences [63,64]. Overall, oxidative stress and metabolic disturbance emerged as nearly universal responses in fish, underscoring the high sensitivity of aquatic vertebrates to combined MP–PAH exposure.

### 5.2. Aquatic Invertebrates

Aquatic invertebrates, including mussels, clams, seaworms, copepods, daphnids, brine shrimp, and corals, were also extensively studied (Table 2). These organism often experience direct contact with MPs in sediments or water columns, making them particularly relevant for evaluating MP-mediated chemical exposure.

#### 5.2.1. Bivalves and Benthic Invertebrates

In mussels (*Mytilus* spp.), exposure to PE, PS, or polypropylene MPs combined with PAHs such as fluoranthene, pyrene, and benzo[a]pyrene resulted in oxidative stress, altered antioxidant defenses, DNA damage, and multilevel physiological toxicity [74], [82,83]. In several studies, MPs acted as vectors enhancing PAH tissue accumulation, supporting the “Trojan horse” effect. However, larger MPs sometimes reduce PAH bioavailability, leading to attenuated toxicity [84]. Manila clams (*Ruditapes philippinarum*) and *Tegillarca granosa* exhibited synergistic immunotoxicity, oxidative stress, and elevated DNA fragmentation when exposed to PAH mixtures with MPs [78,85]. Benthic seaworms (*Hediste diversicolor*) co-exposed to environmental or PVC MPs with benzo[a]pyrene showed significantly increased PAH accumulation and genotoxicity, highlighting strong synergistic interactions [80,86].

#### 5.2.2. Zooplanktons and Reef Organism

Zooplankton and small crustaceans, such as *Daphnia magna* and Arctic copepods, experienced feeding suppression, immobilization, oxidative stress, and reduced fecal pellet production, indicating that MP-PAH interactions can impair key ecological functions [81,87]. Coral studies further revealed oxidative stress and disrupted energy metabolism under co-exposure, suggesting that MP–PAH mixtures pose risks at both organismal and ecosystem levels [88].

### 5.3. Plants, Microorganisms and Cell Lines

Plant, microorganism and cell lines studies, though fewer, provide important insights into terrestrial and aquatic exposure pathways (Table 3).

#### 5.3.1. Higher Plants and Rhizosphere Effects

Crops and aquatic plants such as wheat (*Triticum aestivum*), corn (*Zea mays*), ryegrass (*Lolium perenne*), soybean (*Glycine max*), and water spinach (*Ipomoea aquatica*) exhibited oxidative stress, growth inhibition, photosynthetic impairment, and metabolic disturbances following co-exposure to MPs and PAHs such as phenanthrene and pyrene [73,89,90,95,96]. MP size played a decisive role: smaller MPs induced higher genotoxicity in root tissues, whereas larger MPs often reduced PAH uptake in aboveground tissues by adsorbing contaminants in the soil or water matrix. Meanwhile, MPs altered rhizosphere microbial communities, affecting gene expression of antioxidant enzymes and contributing to chronic toxicity at cellular and molecular levels [73]. These findings indicate that MPs can act both as vectors and buffers in plant systems, significantly influencing contaminant transfer along food chains.

#### 5.3.2. Microalgae and Cell Lines

At the base of the food web, microalgae such as Phaeodactylum tricornutum and Skeletonema costatum exhibited growth inhibition, oxidative stress, reduced photosynthetic efficiency, and altered lipid accumulation under MP–PAH co-exposure [91,97]. In several studies, MPs partially reduced PAH toxicity by sorbing contaminants and lowering effective exposure concentrations. However, at higher MP loads, this mitigating effect diminished, suggesting concentration-dependent interactions. Such responses at the microbial and primary producer level may significantly affect energy transfer and ecosystem productivity.

## 6. Factors Driving Context-Dependent Toxicity Outcomes

The toxicological outcomes of microplastic (MP)–polycyclic aromatic hydrocarbon (PAH) co-exposure vary widely across studies, ranging from synergistic and antagonistic interactions to neutral or mixed biological responses. Rather than reflecting inconsistency, this variability underscores the inherently context-dependent nature of MP-mediated chemical exposure. Differences in particle properties, organismal traits, exposure pathways, temporal dynamics, and experimental design collectively determine whether MPs function as vectors, sinks, or largely passive background particles.

### 6.1. Particle Size, Shape and Surface Characteristics

Particle size is found to be the primary element determining plastic-PAH interactions in aquatic, terrestrial, and plant systems (Table 1). Because of their increased surface area and surface reactivity, nano- and sub-micron plastics regularly exhibit higher sorption capacities per unit mass, which facilitates the transit of PAHs over biological barriers [92,98]. Because of their larger surface area and increased mobility, smaller particles are more able to interact with biological tissues and affect the sorption and transport of contaminants [23]. Therefore, it is essential to distinguish between MPs and nanoplastics in order to understand co-exposure effects. In plant systems exposed to micro- and nanoplastics (MNPs), this size dependence is especially evident. Particles larger than 30 μm exhibited restricted root entrance in hydroponic studies with ryegrass (*Lolium perenne*), but smaller particles (0.1–10 μm) were easily absorbed and carried to shoots [99]. A negative correlation between the translocation of plastics and phenanthrene suggests competitive inhibition during internal transport, and the presence of MNPs decreased the effective concentration and accumulation of PAHs in plant tissues [89]. Additionally, biomarker responses demonstrated that decreased PAH absorption decreased plant toxicity, with the degree of this effect changing with exposure period and particle size. These results show that rather than uniformly increasing exposure, particle size can influence whether plastics limit, redistribute, or alter PAH transport. MPs and nanoplastics should not be regarded as a single category in environmental exposure and risk assessments because smaller and larger plastics can act differently.

On the other hand, numerous studies on aquatic animals do not demonstrate a significant increase in PAH uptake by ingestion of MPs. MPs have been shown to have neutral or only marginally beneficial effects on PAH accumulation in zooplankton, bivalves, and fish, with results primarily determined by feeding behavior, gut residence time, and particle egestion (excretion) rather than internal transport mechanisms [23,81,100].

Toxic reactions become more noticeable when particle size drops to the nanoscale. In comparison to larger MPs, experimental studies in zebrafish embryos show that nanoplastics increase CYP1A induction, oxidative stress, and developmental defects, delay hatching, and improve PAH uptake. On the other hand, bigger MPs (≥10–100 μm) often inhibit absorption or sequester pollutants in the exposure medium, especially in early life stages when particles stay exterior [101,102]. Depending on their size, plastics can have different effects on PAH contamination. While larger plastics may lessen or have no influence on pollution, smaller plastics frequently increase its detrimental effects. This demonstrates that plastic size has a significant impact on how plastics and PAHs damage living things and helps explain why studies occasionally come to different results.

### 6.2. Polymer Type and Affinity

In co-exposure investigations, context-dependent toxicity effects are largely driven by polymer type, which is a major predictor of microplastic–PAH interactions. The strength and reversibility of PAH sorption are determined by variations in polymer chemistry, which affects whether MPs primarily function as vectors that promote transfer to biological tissues or as contaminant sinks that lower bioavailability (Table 1). Because of π–π interactions between the aromatic rings of PAHs and the polymer matrix, polymers with aromatic structures, such polystyrene (PS), typically show greater affinity for PAHs [103]. In both lab and field experiments, where PS particles frequently acquire larger PAH concentrations, this potent sorption capacity has been repeatedly shown [104]. Polyethylene (PE) and polypropylene (PP), on the other hand, interact with PAHs mainly by hydrophobic partitioning since they do not have aromatic functional groups [105]. Despite having relatively lower sorption capabilities than PS, PE and PP are frequently more prevalent in the environment, making them extremely important from an exposure standpoint. PE- and PP-associated PAHs were more easily desorbed and weaker bound in several experiments. This led to a range of biological effects, from greater absorption after ingestion to decreased toxicity when sorption reduced the amounts of freely dissolved PAHs in the exposure medium [106]. Due to their heterogeneous composition and the inclusion of additives, plasticizers, or residual monomers, polyvinyl chloride (PVC) and other less explored polymers exhibit more complex behavior [107]. By changing surface polarity and microplastic aging dynamics, these components can indirectly affect PAH sorption. However, strong conclusions about their significance in co-exposure toxicity are now hampered by a lack of comparative data, indicating a significant study gap.

Polymer-specific PAH affinity is further modulated by environmental aging. UV exposure, oxidation, and biofilm formation are examples of weathering processes that can introduce oxygen-containing functional groups and increase surface roughness, which frequently improves PAH sorption across polymer types [108]. Aging may also decrease sorption selectivity, reducing polymer differences and raising the risk of PAH desorption in changing environmental circumstances [50]. Exposure results are not only determined by polymer identity; rather, PAH availability is shaped by interactions with aging state, particle size, and environmental context.

The durability of plastic–PAH associations and the amount of PAH sorption are both influenced by the kind of polymer, and this has an impact on biological exposure and toxicity. Strongly sorbing polymers can either increase toxicity when ingestion and internal desorption predominate or reduce toxicity by removing PAHs from the surrounding environment. This duality emphasizes the necessity of taking polymer composition into consideration when assessing and comparing ecotoxicological reactions to microplastic–PAH mixtures and helps explain the inconsistent results obtained across co-exposure investigations.

### 6.3. Organismal Traits and Exposure Pathways

Organismal characteristics strongly shape MP–PAH co-exposure outcomes and frequently explain why comparable exposure conditions result in divergent biological responses. Feeding strategy, habitat preference, life stage, gut physiology, and metabolic capacity all influence both the likelihood of encountering MP-bound PAHs and the efficiency of internal transfer.

One of the main factors influencing exposure is feeding behavior. MPs containing sorbed PAHs are more likely to be consumed by organisms that consume particles non-selectively, such as grazing invertebrates, deposit feeders, and filter feeders [94]. When digestive fluids, surfactants, and extended gut residence periods accelerate the release of PAHs from plastic surfaces, ingestion in these species may induce conditions that favor desorption within the gut environment [106]. Therefore, co-exposure often results in increased internal PAH loads as well as increased oxidative stress, genotoxicity, or metabolic disturbance in these organisms. Conversely, under similar environmental conditions, animals with limited particle ingestion or selective feeding techniques may be less exposed.

Exposure paths are further shaped by habitat use. While pelagic animals may come into contact with contaminants predominantly through dissolved phases or trophic transmission [109], benthic organisms living in sediments where MPs and PAHs concentrate are disproportionately exposed by direct touch and ingestion [87,110]. This contrast explains why, even at equal nominal exposure concentrations, benthic invertebrates and demersal fish frequently show higher co-exposure effects than pelagic creatures.

Susceptibility is also influenced by body size and life stage. Because of their immature detoxification mechanisms and permeable biological barriers, early developmental stages like embryos and larvae are more vulnerable to co-exposure [111]. Small MPs and nanoplastics may adhere to or penetrate external tissues during these stages, increasing PAH uptake and interfering with developmental processes [112]. Later in life, improved metabolic capacity and more efficient excretory mechanisms may lessen some effects, though long-term exposure can still have sublethal effects [98].

MPs and PAHs are processed differently by organisms depending on their physiological and biochemical characteristics [113]. In comparison to creatures with low detoxification capacity, species with fast metabolic rates or active biotransformation pathways may quickly metabolize PAHs, changing toxicity profiles [114]. Desorption kinetics and internal exposure can also be influenced by gut anatomy, transit time, and microbiome composition. For instance, quick egestion may restrict internal transfer, whereas extended gut residence durations raise the possibility of PAH release from consumed plastics [115].

Animal and plant-associated systems have quite different exposure pathways. Although plants do not consume MPs, they may come into contact with them through the root-soil or root-water interfaces [116]. The size of the particles affects whether MPs penetrate root tissues and affect internal pollutant transport or stay outside and function mainly as sorptive sinks [117]. PAH absorption and translocation are thus controlled by plant characteristics such as root architecture, transpiration rate, and rhizosphere interactions, which contribute to species-specific and context-dependent results [118].

These organismal characteristics and exposure pathways highlight the need to consider biological context when interpreting microplastic–PAH interactions. Depending on how exposure happens and how pollutants are handled internally, the same microplastic–PAH mixture may increase toxicity in one organism while decreasing or having insignificant effects in another. Therefore, taking organismal characteristics into consideration is crucial for strengthening ecological understanding and explaining variation in co-exposure outcomes.

### 6.4. Exposure Duration, Experimental Design and Neutral Outcomes

Beyond cases of enhanced or reduced toxicity, many studies report neutral or mixed biological responses when organisms are exposed to MPs and PAHs simultaneously. In these situations, the effects observed under co-exposure are like those seen with PAHs or MPs alone, or they vary depending on the endpoint measured, the life stage examined, or the duration of exposure. These findings highlight the inherent complexity of microplastic–PAH interactions and reinforce the idea that their toxicological outcomes are highly context-dependent. Neutral responses are most common when MPs do not noticeably change the fraction of PAHs that organisms can take up. This can occur when sorption and desorption processes are roughly balanced, resulting in no net increase or decrease in effective exposure. Under such conditions, MPs behave primarily as passive background particles, coexisting with PAHs without significantly altering how these chemicals are distributed in the environment or absorbed by organisms [87]. This scenario is often observed when microplastic concentrations are low, particles are relatively large, or contact between organisms and contaminated particles is limited. Mixed outcomes tend to appear when different biological responses move in different directions. For example, organisms exposed to both stressors may show normal survival and growth but display subtle biochemical or molecular changes, such as altered antioxidant activity or shifts in gene expression. While these sublethal effects may not translate into obvious toxicity within the timeframe of an experiment, they could still have longer-term consequences for individual health, reproduction, or population stability [119]. These observations underline the importance of carefully selecting and interpreting endpoints when evaluating co-exposure effects. Life stage and exposure duration further shape whether responses appear neutral or mixed. Short-term experiments may not allow enough time for microplastic-mediated changes in PAH uptake to become apparent, especially in organisms with efficient detoxification or rapid elimination mechanisms [120]. Likewise, adult organisms may tolerate co-exposure without visible effects, while embryos or larvae exposed under the same conditions may respond more strongly [121]. This contrast shows that a neutral outcome in one biological context does not rule out toxicity under another. Experimental design also plays a key role. Simplified laboratory setups, static exposures, or environmentally unrealistic concentrations can mask subtle interaction effects, leading to conclusions that MPs and PAHs do not interact. In contrast, studies that use environmentally relevant conditions, longer exposure periods, or a broader range of biological endpoints often uncover more nuanced responses that cannot be easily classified as purely synergistic or antagonistic [122].

Table 4 highlights the conditions under which microplastic–PAH co-exposure is unlikely to alter toxicological outcomes relative to PAH-only exposure, emphasizing scenarios where neither carrier nor sink mechanisms dominate. Low microplastic concentrations reduce the probability of meaningful interactions between particles, contaminants, and organisms, limiting both PAH sequestration from the dissolved phase and particle-mediated delivery. Under such conditions, PAH exposure is governed primarily by conventional pathways, such as diffusion across respiratory surfaces or dietary uptake from uncontaminated food sources. Larger particle sizes further contribute to neutral outcomes by restricting ingestion and reducing surface area available for PAH sorption. Even when sorption occurs, limited uptake and rapid egestion prevent sufficient contact time for desorption within the gut, minimizing internal transfer. Life stage also plays a critical role, as adult organisms generally possess more developed detoxification and repair mechanisms, including efficient biotransformation and antioxidant defenses, which can buffer modest changes in internal PAH exposure that might arise from microplastic interactions. Short exposure durations similarly constrain the manifestation of microplastic-mediated effects. Carrier-driven enhancement and sink-driven mitigation both require time to influence contaminant partitioning, uptake, and biological response pathways. When exposures are brief, these processes may not reach thresholds necessary to produce measurable toxicological differences. Together, the factors summarized in Table 4 illustrate that neutral outcomes are not contradictory findings but rather reflect exposure scenarios in which the mechanistic conditions needed for MPs to modify PAH toxicity are absent or insufficient.

The neutral and mixed results are evidence of conditions where MPs do not consistently change the amount or pathway of exposure to PAHs in a way that results in evident biological impacts. These findings suggest a dynamic equilibrium between sorption mechanisms, exposure pathways, and organismal characteristics rather than a lack of interaction. To prevent overgeneralization and to more precisely evaluate the ecological significance of microplastic–PAH co-exposure, it is crucial to identify and take into consideration neutral and mixed reactions.

## 7. Biological Synthesis and Implications

### 7.1. Enhanced Toxicity

**Figure 3:**
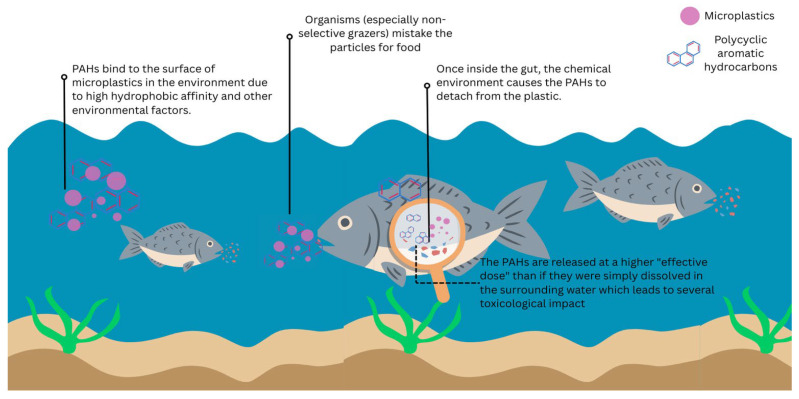
Mechanism of enhanced toxicity during MP-PAH co-exposure. MPs in aquatic systems adsorb PAHs through hydrophobic interactions. Following ingestion by non-selective grazers, gastrointestinal conditions facilitate PAH desorption from the plastic matrix, increasing localized internal exposure and potentially elevating toxicological impact relative to dissolved-phase contaminants.

Many studies report more substantial toxic effects when MPs and PAHs co-occur, especially when MPs facilitate PAH uptake into living organisms. This tends to happen when the characteristics of the particles, especially how organisms are exposed, and the biology of the organisms themselves, work together to promote the release and uptake of PAHs within the body. Under these conditions, organisms can receive a higher internal dose of PAHs than they would from exposure to PAHs alone. Enhanced toxicity is most often observed in studies using small MPs and nanoplastics, which have large surface areas and move easily in water (Table 1). These properties allow them to bind PAHs efficiently and interact closely with biological tissues. In aquatic systems, particularly during sensitive life stages such as embryos and larvae, co-exposure to polystyrene nanoparticles and PAHs has been linked to increased oxidative stress, developmental defects, and effects on the heart and nervous system compared with PAH-only exposure (Table 1 and Table 2). These responses are frequently accompanied by higher PAH concentrations within organisms and the activation of detoxification and stress-related pathways [88], suggesting that MPs can act as effective carriers under certain conditions. Organisms that readily ingest particles are especially vulnerable. Filter-feeding bivalves, deposit-feeding invertebrates, and non-selective grazers commonly show stronger toxic responses during co-exposure. When MPs carrying PAHs are ingested, the digestive environment, which is rich in enzymes and surfactants and characterized by relatively long gut residence times, can promote the release of PAHs from the particles. This process allows contaminants to enter tissues more efficiently than they would in the surrounding water, leading to heightened effects such as DNA damage, immune impairment, and disruptions to energy metabolism [38]. The type of plastic polymer also plays an important role. Polymers with a strong affinity for PAHs, particularly polystyrene, are frequently associated with additive or synergistic toxic effects. These plastics can accumulate PAHs in the environment and later release them after ingestion, effectively transferring contaminants into organisms. When this mechanism coincides with small particle size and sensitive developmental stages, toxicity can be especially pronounced [123,124]. Experimental and environmental conditions further influence whether enhanced toxicity is observed. High microplastic concentrations, limited water volumes, and static exposure systems can amplify carrier effects by increasing contact between organisms and contaminated particles. In such settings, MPs may deliver PAHs at levels higher than those from dissolved exposure alone, producing effects that may not always reflect environmentally realistic scenarios. This underscores the importance of carefully considering exposure context when interpreting experimental results [125]. Figure 3 provides a conceptual illustration of the carrier-mediated toxicity mechanism described above. The figure shows how PAHs readily adsorb onto the surface of MPs in the environment due to hydrophobic interactions, forming contaminant–particle complexes. This can be ingested by organisms, particularly non-selective feeders and particle-feeding species which can mistake MPs for food. Once inside the digestive tract, the chemical environment in the digestive tract promotes the desorption of PAHs from the microplastic surface. This results in localized release of PAHs at a higher effective internal dose than would occur through exposure to dissolved PAHs alone. This vector effect helps explain why co-exposure scenarios frequently lead to elevated internal PAH concentrations and stronger toxicological outcomes, including oxidative stress and developmental and physiological impairments. Further, it synthesizes the interaction between particle properties, organismal feeding behavior and internal exposure dynamics that underpins the enhanced toxicity observed in MP-PAH co-exposure studies.

Enhanced toxicity during microplastic–PAH co-exposure occurs in situations where MPs act as effective vectors that increase internal PAH exposure. However, this outcome is not universal and depends on a specific combination of particle properties, organismal traits, and exposure conditions. Recognizing these contextual factors is crucial for reconciling contrasting findings across studies and for avoiding broad assumptions that MPs inherently amplify chemical toxicity.

### 7.2. Reduced or Mitigated Toxicity

In contrast to studies reporting enhanced toxicity, other research shows reduced or weakened toxic effects during microplastic–PAH co-exposure, particularly when MPs function more as contaminant sinks than as carriers. In these situations, MPs bind PAHs in the surrounding environment, reducing the fraction that remains biologically available. As a result, organisms experience lower internal PAH exposure and milder biological responses than those observed under PAH-only exposure. Toxicity mitigation is reported when MPs significantly reduce the concentration of freely dissolved PAHs in the environment through strong sorption, provided that these contaminants remain tightly bound and do not become bioavailable within the organism’s digestive tract [123]. Larger plastic particles, low ingestion rates, and short gut residence times all reduce the likelihood that PAHs bound to MPs are transferred to organisms [126]. Instead, MPs effectively remove PAHs from the dissolved phase, limiting uptake through respiratory or dermal pathways. This mechanism is especially relevant in pelagic systems, where many organisms are exposed primarily to contaminants dissolved in water rather than through particle ingestion [127]. The type of polymer also influences whether toxicity is reduced.

Aliphatic plastics such as polyethylene and polypropylene tend to bind PAHs through hydrophobic interactions, lowering their freely dissolved concentrations without promoting efficient release within organisms [128]. When ingestion is minimal or absent, these polymers can act as passive sorbents, particularly in systems with high microplastic-to-water ratios or elevated dissolved organic matter that further stabilizes PAH binding. Environmental conditions play a critical role in pollutant-microplastic interactions. Factors such as high salinity can enhance the sorption of PAHs (PAHs) to MPs by reducing their solubility, while dissolved organic matter can compete for binding sites or facilitate transport. Rather than providing a mitigation outcome, these interactions often allow MPs to act as vectors, carrying PAHs into organisms, where conditions such as gut fluids can trigger rapid desorption, thereby increasing biological exposure and toxicity [123]. In more environmentally realistic settings such as flow-through systems or complex matrices like sediments and soils, mitigating effects are often more evident than in simplified laboratory experiments. Reduced toxicity has also been reported in plant-associated systems, where MPs present in soils or growth media influence PAH availability near the root–soil interface. In these cases, relatively large MPs remain outside plant tissues and bind PAHs, lowering their uptake by roots and subsequent transport to aboveground tissues. This indirect pathway highlights how MPs can alter contaminant behavior without serving as direct exposure vectors [129].

**Figure 4:**
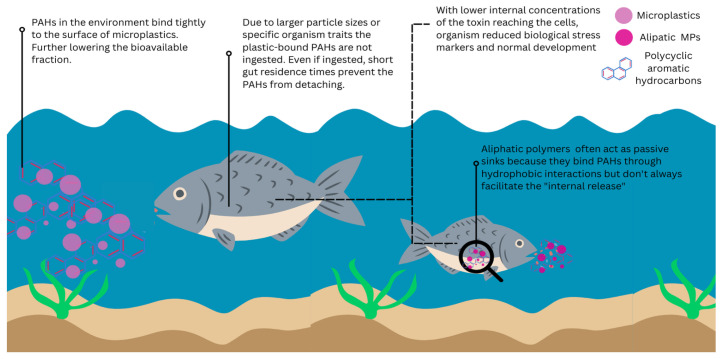
Mechanism of reduced toxicity during microplastic–PAH co-exposure. PAHs in the surrounding water column readily partition onto MP surfaces via hydrophobic interactions, decreasing the freely dissolved fraction. Upon ingestion, particle size, polymer composition, and gastrointestinal residence time govern desorption kinetics. For many aliphatic polymers, strong sorption affinity and limited gut retention restrict PAH release, resulting in reduced internal concentrations compared to dissolved-phase exposure. Consequently, attenuated cellular uptake may lead to lower activation of oxidative stress responses, reduced biomarker expression, and minimal disruption of normal development. This framework highlights the context-dependent role of MPs as passive sinks rather than effective vectors of hydrophobic organic contaminants.

Figure 4 schematically illustrates exposure scenarios in which MPs reduce rather than enhance PAH toxicity by activating primarily as contaminant sinks. PAH in the environment strongly sorbs to microplastic surfaces, decreasing the amount in the surrounding water. When particle characteristics or organismal traits limit ingestion, plastic-bound PAHs are unlikely to be released within the organism. Even when PAHs are ingested, limited desorption in the digestive tract results in lower internal PAH concentrations reaching target tissues and cells. This further highlights the role of aliphatic polymers, which bind PAHs through hydrophobic interactions but do not necessarily facilitate efficient internal release, thereby reducing cellular exposure and downstream biological stress responses. By visually linking strong environmental sorption, limited internal desorption, and reduced biological effects, the figure complements the discussion of mitigation outcomes. It emphasizes how MPs can lower effective PAH exposure under specific particle, organism, and environmental conditions.

MPs are not necessarily protective or beneficial, despite observations of decreased toxicity under co-exposure. Rather, they represent exposure situations when vector-mediated transfer is subordinated to sorption mechanisms. All of these results highlight how MPs can alter the kind and pathway of PAH exposure, increasing or decreasing apparent toxicity depending on the situation. Understanding these circumstances is crucial for resolving contradictory findings in the literature and creating more accurate risk assessments that consider the dual function that MPs play in contaminant dynamics.

Because most studies infer rather than experimentally isolate specific interaction mechanisms, and because multiple mechanisms may co-occur within the same experimental design, mechanistic pathways were synthesized qualitatively rather than quantified.

## 8. Research Gap and Future Perspective

In recent years, there has been a significant increase in research on the combined impacts of PAHs (PAHs) and MPs (MPs). Despite these advancements, there are still significant information gaps that restrict our capacity to assess environmental and health threats in the real world. The existing understanding of MP–PAH interactions is shaped by these limitations, which are not separate but rather interrelated

A significant taxonomic bias is one of the clearest issues. Most MP-PAH research focuses on fish and invertebrates, with relatively little on plants, microbes, and soil-based systems. Understanding MP–PAH interactions at lower trophic levels, where pollutant degradation, sequestration, and transfer are mostly controlled, is hampered by this imbalance. Only a few studies specifically look at MP–PAH interactions among microbial communities and primary producers, even though these groups are crucial in controlling PAH bioavailability and environmental cycling. Because of this, it is yet unknown how cumulative impacts that start at the base of food webs could spread to higher organisms. Expanding research across trophic levels is crucial to filling this gap and assessing the effects at the ecological scale.

Interpretation is also constrained by the types of biological responses measured, as well as by the selection of species. Most of the research focuses on short-term markers such as DNA damage or oxidative stress. Although these endpoints help in the identification of early biological responses, they offer little information about long-term population or ecosystem-level outcomes. Few studies look at recovery after exposure, multigenerational consequences, reproductive success, or chronic exposure. It is challenging to determine whether reported effects are temporary or long-lasting without such information. Life-cycle studies and molecular techniques that connect early cellular responses to long-term ecological repercussions would be beneficial for future research.

These biological uncertainties—PAH interactions themselves—are further exacerbated by the conditional nature of MP. There is growing evidence that co-exposure to MP and PAHs is not always harmful. By sequestering pollutants, MPs can either increase PAH bioavailability and toxicity or decrease harmful pressure, depending on the environmental conditions. This contradictory behavior underscores the need to understand when and why amplification or attenuation occurs and challenges oversimplified notions that MPs always behave as pollution transporters.

Another form of uncertainty is introduced by experimental bias in polymer selection. Despite the fact that environmental plastic contamination is mostly caused by polyethylene (PE), polypropylene (PP), and polyvinyl chloride (PVC), laboratory research primarily uses pure polystyrene (PS) particles. The prominence of PS in experimental designs may exaggerate the toxicity-enhancing impact of microplastics in comparison to real-world circumstances since PS binds PAHs particularly strongly [89,101]. However, despite their growing prevalence in the environment, biodegradable plastics and polymers, including additives, are still not well researched. Environmental relevance would be significantly increased by including a wider variety of polymer types, worn plastics, and realistic mixes.

Exposure conditions themselves are frequently unrealistic, in addition to material selection. Exaggerated carrier effects are more likely in co-exposure investigations because MP and PAH concentrations are frequently much higher than those found in natural settings. Small water volumes, static systems, and brief exposure times may all contribute to an overestimation of the chemical transfer from plastics to living things. Future research should include environmentally relevant concentrations, aged particles, dynamic exposure circumstances, and extended time periods that take natural dilution, particle turnover, and food-web transmission into account to better represent real-world scenarios.

MP-PAH co-exposure research is still not well incorporated into risk assessment and environmental regulation. According to available data, depending on the circumstances, MPs can either increase or decrease PAH toxicity. This casts doubt on the notion that pollution is always made worse by MPs. More standardized study designs and uniform reporting of particle characteristics, chemical behavior, and biological attributes will be necessary to transform this complicated knowledge into useful risk models.

### Methodological Recommendations

Addressing the inconsistencies and uncertainties in MP–PAH co-exposure research requires methodological refinement and greater standardization across studies. One of the most pressing needs is harmonization in experimental design and reporting. Studies should consistently characterize microplastics with respect to polymer type, particle-size distribution, shape, surface area, aging status, and additive composition. Likewise, PAH properties, including molecular weight, hydrophobicity, and sorption behavior, should be explicitly reported. Without standardized physicochemical characterization, cross-study comparison and meta-analysis remain limited. Future research should also incorporate environmentally realistic exposure scenarios. This includes the use of aged or weathered plastics, heterogeneous particle mixtures, and concentration ranges reflecting measured environmental levels rather than worst-case laboratory conditions. Dynamic exposure systems are preferable to static setups because they more accurately simulate dilution, resuspension, and trophic transfer processes. Incorporating natural organic matter and complex matrices would further improve ecological relevance. Another methodological priority is integrating kinetic and mechanistic approaches. Quantifying desorption rates, gut retention times, and chemical partitioning under biologically relevant conditions would clarify whether MPs function predominantly as vectors or passive sinks. Coupling toxicokinetic modeling with biomarker analysis could help link external exposure to internal dose and biological response. Such approaches would allow clearer differentiation between particle-driven effects and chemical-driven toxicity. Multi-endpoint and multi-scale assessments are also recommended. Beyond short-term oxidative stress and genotoxicity markers, studies should include endpoints related to reproduction, growth, behavior, immune function, and multigenerational outcomes. The integration of omics-based techniques (transcriptomics, proteomics, metabolomics) with conventional toxicological endpoints may help bridge molecular responses and population-level consequences.

Finally, improved transparency and data reporting standards are essential for regulatory translation. Detailed documentation of experimental conditions, analytical verification of exposure concentrations, and reporting of both positive and null findings would reduce publication bias and enhance reproducibility. Collaborative, interlaboratory studies and standardized testing frameworks could further strengthen the reliability of MP–PAH co-exposure research and facilitate its incorporation into environmental risk assessment models.

## 9. Conclusions

Recent evidence indicates that interactions between microplastics (MPs) and polycyclic aromatic hydrocarbons (PAHs) are highly context-dependent rather than uniformly harmful. Under certain environmental and biological conditions, MPs may enhance PAH toxicity by facilitating sorption, ingestion, and internal desorption, leading to oxidative stress, inflammation, and immunological impairment. In other cases, MPs may reduce apparent toxicity by temporarily sequestering PAHs and limiting their bioavailability. This variability demonstrates that MP–PAH interactions cannot be explained by a simple vector hypothesis. Instead, MPs function as dynamic modifiers of contaminant fate and biological exposure. The direction and magnitude of effects depend on polymer type, particle size, environmental aging, exposure pathway, concentration, and organism-specific traits. From a regulatory perspective, these findings indicate that risk assessment frameworks should move beyond single-contaminant evaluations and explicitly consider microplastics as dynamic modifiers of chemical exposure rather than universal toxicity amplifiers. Incorporating mixture-based approaches, environmentally realistic exposure scenarios, and life-stage sensitivity into policy-relevant assessments will improve the accuracy of ecological risk predictions for microplastic-associated organic pollutants.

These findings have important implications for environmental chemistry and regulatory risk assessment. Risk evaluation frameworks should move beyond single-contaminant approaches and explicitly consider MPs as modifiers of chemical exposure within mixture scenarios. Incorporating environmentally realistic concentrations, chronic exposure designs, and life-stage sensitivity into future assessments will improve the ecological relevance and predictive power of risk evaluations for MP-associated organic pollutants.

## Figures and Tables

**Table 1:** Toxicological effects of MPs and PAHs exposed to aquatic vertebrates.

Species	MP Type and Concentration	PAH Type and Concentration	Toxicological Effects			Reference
			MP Only	PAH Only	Co-Exposure Effects	
*Danio rerio* (*zebrafish*)	PS, 5 × 10^−5^ mg/mL	B[a]P, 2.1 × 10^−5^	Regulation of oxidative stress genes in liver	Strong oxidative stress	Small MPs enhanced PAH accumulation	[53]
*Danio rerio* (*zebrafish*)	PS, 1 mg/mL	Phenanthrene, 0.00001–0.00005 mg/mL	Mild developmental delay	Reduced hatching; edema; lipid peroxidation	Synergistic developmental toxicity	[54]
*Danio rerio* (*zebrafish*)	0.00001 mg/mL	Phenanthrene, 2 × 10^−13^ mg/mL	No significant biomarker effects	Increased Cytochrome P450 activity and metabolic rate.	MO_2_ increased by 70% (not statistically significant)	[55]
*Danio rerio* (*zebrafish*)	PS, 0.01 mg/mL	B[a]P	Slight growth reduction; low oxidative stress	Strong oxidative stress	MPs acted as vectors, enhancing toxicity	[56]
*Danio rerio* (*zebrafish*)	PS, 0.02 mg/mL	B[a]P, 0.0001 mg/mL	No mortality; no cardiotoxicity	DNA damage; lipid peroxidation	Small MPs enhanced cardiotoxicity	[57]
*Danio rerio* (*zebrafish*)	PS, 0.0001–0.001 mg/mL	Phenanthrene, 0.0001–0.005 mg/mL	Developmental abnormalities; mortality	Mild oxidative stress	No mortality; no cardiotoxicity	[58]
*Danio rerio* (*zebrafish*)	PS, 0.001–0.005 mg/mL	Fluoranthene, 0.001 mg/mL	DNA damage; lipid peroxidation	DNA damage; oxidative stress	Additive genotoxicity	[59]
*Danio rerio* (*zebrafish*)	PE, 0.01–0.04 mg/mL	HPAHs	Neurotoxicity; oxidative stress	Strong oxidative stress	MPs increased PAH bioavailability	[60]
*Danio rerio* (*zebrafish*)	PS, 0.003 mg/mL	Phenanthrene, 0.0002 mg/mL	DNA damage; disturbed amino acid pathways	Strong oxidative stress	Caused oxidative stress, increased immunity gene expression, increased immunity gene expression altered gut microbiota	[61]
*Danio rerio* (*zebrafish*)	PE, 1 × 10^−5^ mg/mL	Anthracene, 0.02 mg/mL	Weak toxicity	Reduced swimming; CYP1A induction	Small MPs enhanced PAH accumulation	[62]
*Oryzias melastigma*	PS, 0.0002 mg/mL	Phenanthrene, 5 × 10^−5^ mg/mL	Low bioavailability	Gut dysbiosis; oxidative stress	Transgenerational reproductive toxicity	[63,64]
*Lates calcarifer*	PS, 100 particles/L	Pyrene, 100 nM	Minor behavioral effects	Reduced feeding rate	Synergistic behavioral impairment	[65]
*Lates calcarifer*	PE, 0.0001–0.001 mg/mL	Benzo(a)pyrene	Mild oxidative stress	Behavioral alteration	Synergistic negative effects	[66]
*Carassius auratus*	PS, 10–100 beads/L	Benzo(a)pyrene	Strong oxidative stress	Antioxidant disruption	Multilevel toxicity	[67]
*Oncorhynchus mykiss*	PS, 0.005 mg/mL	3-NBA	No cytotoxicity	DNA damage	Reduced cell viability	[68]

**Table 2:** Toxicological effects of MPs and PAHs exposed to aquatic invertebrates.

Species	MP Type and Concentration	PAH Type and Concentration	Toxicological Effects			Reference
			MP Only	PAH Only	Co-Exposure	
*Mytilus galloprovincialis*	PS, 0.01 mg/mL	Fluoranthene, 0.0001–0.001 mg/mL	Increased ROS	DNA damage	Trojan-horse genotoxicity	[74]
*Mytilus galloprovincialis*	PP, 0.001 mg/mL	Pyrene, 0.00005 mg/mL	Mild oxidative stress	Strong oxidative stress	Multilevel toxicity	[75]
*Mytilus coruscus*	PS, 0.00003 mg/mL	Phenanthrene, 0.00005 mg/mL	Immune modulation	Genotoxicity	Synergistic hemocyte stress	[76]
*Mactra veneriformis*	PS, 0.001 mg/mL	Phenanthrene, 0.00002–0.00005 mg/mL	Oxidative stress; size-dependent enzyme inhibition	Dose-dependent oxidative stress; antioxidant induction → inhibition	Joint exposure amplified toxicity; phenanthrene-dominated effects; larger MPs intensified inhibition of SOD and GST	[77]
*Tegillarca granosa*	PS, 194 ng/g	Mixture of 16 PAHs	Immune suppression	Oxidative stress	Synergistic immunotoxicity	[78]
*Daphnia magna*	PS, 0.01–0.1 mg/mL	HPAHs	Size-dependent oxidative stress	Acute toxicity	Increased immobilization	[79]
*Hediste diversicolor*	PVC, 10–50 mg/kg	Benzo(a)pyrene	Mild stress	Genotoxicity	Increased PAH accumulation	[80]
*Arctic copepods*	PE, 20 MPs/mL	Phenanthrene, 1 µL oil L^−1^	No mortality	Reduced fecal pellets	Feeding suppression	[81]

**Table 3:** Toxicological effects of MPs and PAHs exposed to plants, microorganisms and cell lines.

Species	MP Type and Concentration	PAH Type and Concentration	Toxicological Effects			Reference
			MP Only	PAH Only	Co-Exposure	
*Zea mays* L.	PE, D550 (550 µm) and D250 (250 µm)	Phenanthrene, 0.1 mg/mL	Root oxidative stress, increased protease and catalase activity	Accumulation; reduced root activity, Root oxidative stress	MP decreased PHE accumulation in leaves by 64.9–88.5%	[73]
*Lolium perenne*	PS, 0.1–10 μm	Phenanthrene, 0.0005–0.005 mg/mL	Minimal phytotoxicity	Chlorophyll loss	Growth inhibition	[89]
*Ipomoea aquatica*	MPs, 0.1–10 μm	Pyrene, 100 nM	Mild oxidative stress	Lipid peroxidation	Increased PAH uptake	[90]
*Phaeodactylum tricornutum*	uPVC, 1 mg/mL	Phenanthrene	Growth inhibition	Oxidative stress	MPs reduced PAH toxicity	[91]
*Skeletonema costatum*	PS, (2 μm)	Pyrene, 0.0005–0.002 mg/mL	Mild oxidative stress	Growth inhibition	Synergistic stress	[92]
*Rat liver cells*	PS, 0.5 μm	B[a]P, 1 mg/kg	Oxidative stress	Inflammation	Amplified oxidative damage	[93]
*Skeletonema costatum*	PS, n.s.	B[a]P	Mechanical irritation	Oxidative stress	Inflammatory amplification	[94]
*RTgill-W1 cells*	PS, 5 µg/mL	3-NBA	No cytotoxicity	DNA damage	Decreased viability	[68]

Note: n.s. = not specified.

**Table 4:** Factors contributing to neutral toxicological outcomes during microplastic–PAH co-exposure.

Factor	Why It Leads to a Neutral/Mixed Outcome
Low Concentration	Not enough particles to significantly act as a sink or a vector.
Large Particle Size	Reduced surface area for sorption and lower likelihood of ingestion.
Adult Life Stages	Higher resilience and more robust metabolic pathways compared to larvae.
Short Duration	The exposure time is too brief for “carrier effects” or “sequestration” to manifest.

## Data Availability

No new data were created or analyzed in this study. Data sharing is not applicable to this article.
